# The Role of Prenatal Melatonin in the Regulation of Childhood Obesity

**DOI:** 10.3390/biology9040072

**Published:** 2020-04-05

**Authors:** Dmitry O. Ivanov, Inna I. Evsyukova, Gianluigi Mazzoccoli, George Anderson, Victoria O. Polyakova, Igor M. Kvetnoy, Annalucia Carbone, Ruslan A. Nasyrov

**Affiliations:** 1 Saint-Petersburg State Pediatric Medical University, 194100 St. Petersburg, Russia; doivanov@yandex.ru (D.O.I.); vopol@yandex.ru (V.O.P.); rrmd99@mail.ru (R.A.N.); 2 Ott Research Institute of Obstetrics, Gynecology and Reproductology, 199034 St. Petersburg, Russia; eevs@yandex.ru; 3 Department of Medical Sciences, Division of Internal Medicine and Chronobiology Laboratory, Fondazione IRCCS Casa Sollievo della Sofferenza, 71013 San Giovanni Rotondo, Italy; annalucia.carbone@gmail.com; 4 CRC Scotland & London, London E14 6JE, UK; anderson.george@rocketmail.com; 5 Saint-Petersburg State University, University Embankment 7/9, 199034 St. Petersburg, Russia; igor.kvetnoy@yandex.ru

**Keywords:** melatonin, obesity, prenatal, circadian, postnatal, development, metabolism, mitochondria, comorbidity, gut

## Abstract

There is a growing awareness that pregnancy can set the foundations for an array of diverse medical conditions in the offspring, including obesity. A wide assortment of factors, including genetic, epigenetic, lifestyle, and diet can influence foetal outcomes. This article reviews the role of melatonin in the prenatal modulation of offspring obesity. A growing number of studies show that many prenatal risk factors for poor foetal metabolic outcomes, including gestational diabetes and night-shift work, are associated with a decrease in pineal gland-derived melatonin and associated alterations in the circadian rhythm. An important aspect of circadian melatonin’s effects is mediated via the circadian gene, BMAL1, including in the regulation of mitochondrial metabolism and the mitochondrial melatoninergic pathway. Alterations in the regulation of mitochondrial metabolic shifts between glycolysis and oxidative phosphorylation in immune and glia cells seem crucial to a host of human medical conditions, including in the development of obesity and the association of obesity with the risk of other medical conditions. The gut microbiome is another important hub in the pathoetiology and pathophysiology of many medical conditions, with negative consequences mediated by a decrease in the short-chain fatty acid, butyrate. The effects of butyrate are partly mediated via an increase in the melatoninergic pathway, indicating interactions of the gut microbiome with melatonin. Some of the effects of melatonin seem mediated via the alpha 7 nicotinic receptor, whilst both melatonin and butyrate may regulate obesity through the opioidergic system. Oxytocin, a recently recognized inhibitor of obesity, may also be acting via the opioidergic system. The early developmental regulation of these processes and factors by melatonin are crucial to the development of obesity and many diverse comorbidities.

## 1. Introduction

The rise in childhood obesity is widely recognized as major worldwide health issue [1], not only in western cultures but also in developing countries [2,3]. Unfavourable intrauterine conditions contribute to offspring obesity risk, including when associated with maternal conditions, such as obesity, diabetes, metabolic syndrome, or chronic disorder of three or more functional systems (cardiovascular, gastrointestinal, immune, etc.) as well as from pregnancies being complicated by chronic placental deficiency, preeclampsia, or gestational diabetes [4,5,6]. Childhood obesity can be associated with neonates born heavy for height as well as light for height with associated “catch-up” excessive weight gain [7,8,9]. Internal obesity in the first postnatal months increases the risk of the later development of type 2 diabetes, metabolic syndrome, and cardiovascular and nervous system pathologies [10,11,12]. Such data highlights the importance of the prenatal period in modulating obesity predisposition as well as in indicating the important role that pregnancy has as an “environmental sampling” period for adaptive development. Postnatal factors, such as formula-feeding vs breastfeeding, also contribute to obesity risk as associated health consequences, including an array of childhood and adult cancer. A number of general biological processes have been proposed to underpin the prenatal adaptations that heighten offspring obesity risk, including oxidative stress; epigenetic processes; glucocorticoid effects; as well as the actions of neuroactive steroids, somatolactogenes, and related peptides, such as insulin-like growth factor (IGF-1) and oxytocin [13,14,15]. In each of these mechanisms, we can see a certain role of the melatonin hormone as a key element, the absence or lack of which determines the activation of processes leading to obesity “programming”. This article reviews the role of melatonin in the prenatal development of obesity risk, highlighting its powerful role in the regulation of mitochondrial metabolism.

## 2. Melatonin, Metabolism, and Mother–Placenta–Fetus Interface

Melatonin is classically known for its role in the regulation of the circadian rhythm following its nighttime release by the pineal gland. Melatonin is a powerful antioxidant, an inducer of endogenous antioxidants, and an anti-inflammatory and optimizer of mitochondrial function. Melatonin effects can be via the melatonin receptors, primarily MT1 and MT2 receptors, as well as via nonreceptor effects. Melatonin is generally regarded as amphiphilic, being able to diffuse through the extramembrane spaces as well as through the bilipid cell membrane. Exogenous melatonin tends to gather around intracellular organelles, especially mitochondria, where it can be actively taken up by transporters [16]. Recent data indicates that melatonin is produced by all mitochondria-containing cells, including high levels of production in the gut and placenta. Recent work also shows melatonin to be produced within mitochondria, where it may act to regulate metabolism, sirtuins, endogenous antioxidants, and the mitochondrial antioxidant/oxidant ratio [17]. Melatonin is therefore a powerful regulator of the mother–placenta–fetus interface [18,19,20,21,22].

Given the presence of G-protein-related receptors in fetal tissues, melatonin directly modulates adrenal cortisol production and lipolysis in brown adipose tissue [23,24]. Genetic and epigenetic factors that act prenatally can modulate processes associated with obesity, including hypothalamic neuropeptides and glucocorticoid receptors [25,26,27,28,29]. Epigenetic modifications in the histone (H3K4) structure of the hepatic insulin-like growth factor (IGF) lead to increase of IGF-1 levels in the blood of delayed fetuses, which “programs” their catch-up growth in the first months of life [30,31]. Melatonin and other protective factors inhibit the induction and effects of such epigenetic changes.

Many of melatonin’s circadian effects are mediated via the induction of the circadian gene, BMAL1, including effects on mitochondrial metabolism. The circadian genes, Clock and BMAL1, are important not only to the circadian regulation of mitochondrial metabolism but also to wider energy regulation, including daytime glucose and triglycerides levels [32] as well as lipid synthesis, adipogenesis [33], carbohydrate, and adipose metabolism [34]. Melatonin also has insulin-like hypoglycemic, anabolic and anti-cholesterol effects [35], with numerous studies showing the functional interactions of melatonin, insulin, and glucagon [36,37], including in the pancreatic circadian rhythm of glucose production [38]. Melatonin’s effects in the pancreas and in other tissues and organs are partly mediated via circadian genes [39]. As a consequence, melatonin shows a negative correlation with circulating insulin levels, with the suppression of pineal melatonin, as in night-shift work, leading to hyperinsulinemia, insulin resistance, and hyperleptinemia as well as a significant decrease in glucose transporter (GLUT)4 levels, which are characteristic of type 2 diabetes [40,41]. The prolonged exposure to artificial light that is characteristic of most cultures also drives down melatonin levels in correlation with an increase in abdominal obesity, arterial hypertension, and lipid and carbohydrate metabolism disorder [42,43].

Circadian melatonin production increases over pregnancy, coupled to rising levels of placental melatonin as the placenta grows [44,45]. Pineal melatonin synchronizes the circadian rhythms of trophoblasts and endothelial tissue [46], which coincides with similar clock genes expression in the fetus, thereby determining the development of fetal adaptive metabolic processes and normal growth [47,48]. It is assumed that the fetal circadian rhythm becomes evident in the fetus following the appearance of melatonin receptors that are transferred from the mother at the earliest stages of pregnancy [49,50]. This is supported by data in nonhuman primates [51], with continuous light exposure in pregnant rats disrupting the rhythmic expression of clock genes in fetus [52]. Although the fetal pineal gland produces melatonin, its rhythm is determined by the mother’s pineal melatonin production, which is important to the morphological and functional development of suprachiasmatic nucleus (SCN) and pineal gland [53] and other rhythmic body systems, including cardiac, temperature, and cortisol [54].

Intrauterine growth retardation (IUGR) occurs in the absence of a melatonin circadian rhythm in the maternal blood plasma during the second half of pregnancy. This is accompanied by the delayed development of the SCN [55] and the corticosterone rhythm [56]. This would suggest that the fetal SCN is like a peripheral oscillator that is driven and developed by maternal pineal melatonin. This prepares the fetus for the postnatal integration of endogenous biorhythms driven by light acting on the retinohypothalamic tract [57]. This suggests that circadian entrainment is a core aspect of pregnancy as a period of “environmental sampling”.

Clearly, the loss of such prenatally programmed circadian information would have important consequences for the fetus. The absence or suppression of melatonin’s circadian production in pregnant women, as can occur with maternal obesity, metabolic syndrome, endometriosis, and polycystic ovary syndrome as well as in pregnancies complicated by preeclampsia, chronic placental insufficiency, or night work [58,59,60,61,62], will disrupt SCN and circadian rhythm development, with consequences for metabolism [63,64]. This is important to a wide array of body systems, including the immune system, which is regulated over the circadian rhythm and is an important driver of a host of diverse medical conditions [65].

Under optimal conditions, the circadian rhythm is reinforced in the newborn via melatonin transfer through breast milk, especially colostrum [66,67,68]. Formula-feeding can therefore contribute to the desynchronization of metabolic processes, imbalances in energy exchange, and excessive weight gain [69,70,71]. Such data has contributed towards a drive to have a nighttime formula feed that contains melatonin, which may be important for the high percentage of mothers who do not exclusively breastfeed [72], especially as this may be important to the development of the gut–brain axis [73]. This is supported by a recent retrospective study, where breast-feeding by nonobese mothers for 10–12 months showed good infant outcomes, including measures of weight, length, and colic. This contrasts with the outcomes of mothers with obesity/obesity complications and who combined early maternal milk/later complimentary feeding with formula-feeding of their infants with all infants having colic [74]. The absence of a circadian melatonin rhythm in obese mothers was also associated no circadian melatonin rhythm in their offspring, whilst the offspring of nonobese mothers showed a low but significant circadian melatonin rhythm from the 3rd postnatal day [74]. Such data highlights the importance of the melatonin circadian rhythm over pregnancy as well as postnatally [75,76,77].

This has relevance to other aspects of pregnancy, including development of the immune system, mitochondrial function, gut microbiome, and gut barrier integrity as well as to the effects of melatonin on ceramide levels and to melatonin’s effects being mediated not only via BMAL1, but also the alpha 7 nicotinic receptor and opioidergic system. All of these processes and factors are associated with obesity and metabolic regulation.

## 3. Placenta and Immune Cells

Preeclampsia and many other pathophysiological and physiological processes in the placenta are intimately linked to changes in the activity and phenotypes of immune cells, including natural killer (NK) cells and macrophages, which are the most common decidual leukocytes [78]. Both NK cells and macrophages show phenotypic changes over the course of pregnancy [78,79]. Both of these immune cell types are regulated by melatonin as well as melatonin-induced BMAL1 and alpha 7 nicotinic acetylcholine receptor (α7nAChR), with autocrine melatonin acting to switch macrophages from an M1-like pro-inflammatory phenotype to an M2-like phenotype [80]. As immune cells are powerful controllers of the survival and function of other cells, including placental, such impacts of melatonin on immune cells are important to placental pathophysiology [81]. It is also of note that an obesogenic diet is associated with significant alterations in placenta-associated immune cells [82], being one mechanism whereby maternal obesity impacts on offspring outcomes. Melatonin is a significant regulator of the poor outcomes associated with maternal obesity and an obesogenic diet [83], with effects partly mediated via alterations in the placenta’s regulation by immune cells.

## 4. Immune Cells and Mitochondria

The phenotype of immune cells are driven by alterations in mitochondrial metabolic function, primarily mediated by glycolysis in a reactive state and oxidative phosphorylation (OXPHOS) in a more quiescent, M2-like state [84]. This shift in phenotype is evident over the circadian rhythm and is the essence of the immune-pineal axis [85], with pineal melatonin shifting immune cells to a quiescent phenotype unless suppressed by the need for an ongoing immune response, as indicated by an increase in pro-inflammatory cytokines [86]. Circadian melatonin therefore has similar effects to those of autocrine melatonin in the regulation of immune cell phenotype [80], allowing both circadian and cellular melatonin to be important determinants of immune cell function. Variations in melatonin production, both circadian and local, thereby underpin alterations in immune cell mitochondrial function that can drive significant changes in the placenta and developing foetus.

Melatonin effects on mitochondrial function, both direct and via BMAL1, are mediated by an increase in the conversion of pyruvate to acetyl-CoA, thereby increasing ATP production by the tricarboxylic acid (TCA) cycle and OXPHOS [87]. This requires the disinhibition of the pyruvate dehydrogenase complex (PDC), with PDC driving the conversion of pyruvate to acetyl-CoA. As acetyl-CoA is also a necessary co-substate for AANAT and the activation of the mitochondrial melatoninergic pathway, circadian melatonin acts to upregulate mitochondrial melatonin and thereby increase levels of sirtuins and endogenous antioxidant enzymes, including superoxide dismutase (SOD)2 [88,89]. As such, local and circadian melatonin can significantly determine the changing immune responses required in placenta-regulating immune cells via impacts on mitochondrial function. The effects of circadian melatonin, via BMAL1 and possibly the α7nAChR, include the upregulation of mitochondrial melatoninergic pathway.

The initiation of the melatoninergic pathway requires the stabilization of AANAT by different 14-3-3 isoforms; 14-3-3 is evident in mitochondria, with its cellular levels being decreased by factors that are associated with suboptimal placental function and poor foetal outcomes, including increased ceramide levels and the microRNAs, miR-7, miR-375, and miR-451 [90]. This would suggest that factors that increase ceramide and these 14-3-3-regulating miRNAs would be associated with poor outcomes, at least in part, via suboptimal mitochondrial melatoninergic pathway activation and the consequences that this has for cellular function, including immune cell function. As melatonin is produced in all body cells, including placental and foetal, alterations in the regulation of the melatoninergic pathway will be relevant to the survival and function of all cells. However, its most relevant impact on human pathophysiology seems predominantly via alterations in the regulation of mitochondrial function, especially in immune cells.

## 5. Melatonin and Mitochondria

Mitochondria are also important to trophoblast function, with alterations in trophoblast mitochondrial function evident in conditions associated with offspring obesity, including preeclampsia and gestation diabetes [91,92]. The decrease in trophoblast mitochondrial respiration in gestational diabetes seems mediated by an increase in ceramide [92], with ceramide also shown to increase mitochondrial fusion and mitophagy in preeclampsia trophoblasts [93]. As to whether this is mediated by ceramide’s inhibition of 14-3-3 and therefore the mitochondrial melatoninergic pathway requires investigation. In contrast, preeclampsia may be associated with an increase in trophoblast mitochondrial respiration, although with a decrease in respiratory reserve capacity [94]. Preclinical studies indicate that there may differential effects on mitochondrial function in different placental regions [91]. As well as immune cells, trophoblast mitochondrial function regulation by local and circadian melatonin are clearly relevant to placental changes that increase offspring obesity risk.

## 6. Maternal Gut Microbiome and Pregnancy

There is increasing interest in the role of the gut dysbiosis and associated gut permeability in a wide array of diverse medical conditions [95,96], including in preeclampsia [97]. A decrease in gut microbiome diversity is associated with a significant drop in levels of the gut microbiome-derived short-chain fatty acid, butyrate [97]. Treatment of a preclinical preeclampsia model with butyrate lowered blood pressure, suggesting a role for butyrate in the regulation and treatment of preeclampsia. Clinical and preclinical data show gestational diabetes and intrauterine growth retardation to also have alterations in the gut microbiome, with probiotics shown to have some efficacy in the management of gestational diabetes patient outcomes [98].

Butyrate has a number of effects that are relevant to its efficacy in such a wide array of medical conditions. Butyrate is readily taken up by intestinal epithelial cells, which maintains the gut barrier. Butyrate is also readily transported across intestinal epithelial cells into the general circulation where it can have impacts on central and body-wide systems. Butyrate is a histone deacetylase (HDAC) inhibitor and therefore a powerful epigenetic regulator, including the rapid and marked upregulation of the μ-opioid receptor [99]. Butyrate also dampens immune and glia cell reactivity, with effects that seem mediated by its optimization of mitochondrial function, including the upregulation of pyruvate dehydrogenase complex (PDC) and therefore of oxidative phosphorylation (OXPHOS) and the tricarboxylic acid (TCA) cycle. Butyrate also increases the activation of the melatoninergic pathway, as shown in intestinal epithelial cells [100], with butyrate able to decrease gut permeability, thereby preventing the effects of circulating lipopolysaccharides (LPS) on immune and other body functions. Butyrate may also regulate the melatoninergic pathway via its conversion of ceramide to glucosyl-ceramide, thereby preventing ceramide’s inhibition of 14-3-3 [101]. Data in primates shows butyrate to significantly modulate trophoblast and placenta development [102].

It is widely accepted that alterations in the gut–liver and gut–brain axes are important to paediatric and adult obesity, with decreased butyrate an important aspect of this [103]. The above would suggest that alterations in butyrate availability prenatally may also be relevant to the early developmental etiology of obesity and its associated complications in children and adults.

The role of butyrate-induced mitochondrial melatonin and OXPHOS will be important to determine in the human placenta as well as its influence on the shift in immune cell activity that are crucial to placenta and foetal development. It has recently been proposed that such processes can drive changes in the foetal gut and associated immune cells, especially γδT cells, with consequences for infant post-natal development [81].

It should also be noted that it is not only recognized prenatal medical conditions, such as IUGR and gestational diabetes, that increase offspring obesity. A number of studies show a variety of prenatal stressors to also have such impacts on human offspring [104]. The effects of different stressors is partly mediated by an increase in corticotropin-releasing hormone in the hypothalamus and amygdala, which then acts on mucosal mast cells to increase tumor necrosis factor α (TNFα), which then increases gut permeability and contributes to gut dysbiosis [105]. As such, some of the effects of prenatal medical conditions may be mediated by the stress associated with the symptoms and diagnosis, with consequences driven partly by gut dysbiosis/permeability. Within such a context, the gut and body mitochondria form two important hubs, with their interactions modulated by the levels of melatonin availability [89].

Overall, alterations in the gut microbiome and butyrate production are intimately linked to the prenatal etiology and postnatal pathophysiology of obesity via processes in a number of different body systems and organs but with communal effects that seem mediated by the mitochondrial melatoninergic pathway.

## 7. Melatonin and the Alpha 7 Nicotinic Receptor

Melatonin effects may also be via its induction of the α7nAChR, which is positively regulated in a circadian manner by the pineal hormone [106]. As well as being present on the plasma membrane, the α7nAChR is expressed on mitochondria, where it acts to suppress apoptotic processes [107]. Some of the effects attributed to melatonin, including in the regulation of gut permeability, are mediated by melatonin increasing vagal nerve ACh that then activates the α7nAChR [108]. The α7nAChR also acts to dampen immune and glia cell pro-inflammatory activity and to shift cells to a phenotype associated with OXPHOS and a more quiescent phenotype. As to whether this is driven by an α7nAChR-mediated disinhibition of PDC and upregulation of the TCA cycle and the mitochondrial melatoninergic pathway requires investigation. This could suggest that the α7nAChR, like BMAL1, mediates pineal melatonin’s induction of mitochondrial melatonin via PDC disinhibition [109]. 

There is a decrease in placental α7nAChR mRNA and protein in women with preeclampsia [110], indicating that this would be correlated with the decrease in placental melatonin production that is also evident in this condition [111]. Preeclampsia is also associated with a decrease in pineal melatonin, which may be especially evident in preeclamptic women with non-dipping nighttime blood pressure [112]. Melatonin’s positive regulation of the α7nAChR may also directly regulate obesity, since α7nAChR agonism is associated with decreased food intake [113]. Alterations in α7nAChR levels and activation are relevant to other aspects of obesity, including in modulating the effects of central insulin on hepatic gluconeogenesis. Central insulin effects are mediated via the vagal nerve ACh acting on to the α7nAChR of Kupffer cells and hepatic macrophages, which are dysregulated in high fat diet and insulin resistance and drive many hepatic pathophysiological changes in obesity [114]. Macrophage α7nAChR activation can prevent obesogenic impacts on adipocytes [115], whilst the α7nAChR is decreased in the white adipose tissue of obese individuals [116]. Such data indicates a role for the α7nAChR in the regulation of obesity via impacts on different cells and in different tissues, indicating that its regulation by variations in melatonin availability may be important to different aspects of the pathoetiology and pathophysiology of obesity.

The α7nAChR can be negatively regulated by its uniquely human duplicant dupα7 (CHRFAM7A), suggesting that the differential genetic and epigenetic regulation of α7nAChR and dupα7 will determine many of melatonin’s effects. The relevance of the differential regulation of α7nAChR and dupα7 in the placenta, foetus, and placenta-associated immune cells will be important to determine, including how this modulates the levels and effects of melatonin.

## 8. Melatonin and the Opioidergic System

Alterations in the opioidergic system, especially via reward regulation, are intimately associated with food intake and its dysregulation in obesity [117]. The opioidergic system is also integral to the associations of depression/mood with alterations in food intake [118]. Preclinical and human data shows the μ-, δ-, and κ-opioid receptors regulate metabolic response to diet [119,120,121]. The opioidergic system is also evident in the placenta and is integral to the regulation of immune responses [118]. Activation of the μ-opioid receptor is classically associated with reward, with a decrease in μ-opioid receptor levels proposed to drive food intake in obese individuals [119]. The activation of the κ-opioid receptor, especially in the amygdala, can be associated with dysphoria in humans [122], with κ-opioid receptor inhibition decreasing food intake in obesity [123]. The knockout of dynorphin, the endogenous κ-opioid receptor agonist, reduces fat mass and increases weight loss in mice [124], indicating a role for dysphoria in the regulation of food intake, commonly referred to as “comfort eating”.

Melatonin is a significant regulator of the opioidergic system, including positively regulating the circadian levels of β-endorphin, the endogenous μ-opioid receptor agonist, as well as decreasing κ-opioid receptor levels, reviewed in Reference [118]. It is also of note that gut microbiome-derived butyrate epigenetically upregulates the μ-opioid receptor [99], suggesting that the optimization of butyrate and melatonin will upregulate the μ-/κ-opioid receptor ratio levels and activity, with consequences for food intake in association with mood regulation. As such, the impact of circadian and local melatonin regulation of prenatal processes in the modulation offspring obesity may be intimately linked to changes in the offspring’s opioidergic system. Clearly, the role of melatonin and maternal-derived butyrate in the regulation of the placental and foetal opioidergic systems require further investigation.

Alterations in the activation of the opioidergic system may also be driven by oxytocin. Oxytocin is classically associated with parturition and mother–baby bonding. However, recent data shows oxytocin to significantly suppress obesity, indicating wider roles in the regulation central and systemic processes [125]. It is also of note that oxytocin is a positive allosteric modulator of the μ-opioid receptor [126], thereby linking oxytocin effects to data showing the role of the μ-opioid receptor in obesity as well as in attachment, nociception, and reward. As melatonin can regulate hypothalamic oxytocin production in rodents [127], it requires investigation as to whether variations in circadian and local melatonin production are relevant to the modulation and development of the oxytocin system in the placenta and foetus.

## 9. Conclusions

The control of the developing fetal circadian rhythm by maternal melatonin is clearly an important aspect of the development of a wide array of human conditions, including obesity and metabolic dysregulation. Many factors can influence maternal melatonin levels during pregnancy, including night-shift work, night-light exposure, maternal depression as well as an array of genetic and epigenetic factors. The crucial effects of pineal melatonin may be mediated, via BMAL1 and possibly the α7nAChR and opioidergic system, on the mitochondrial melatoninergic pathway and therefore on mitochondrial metabolism. This may be of particular importance to the developing immune system, both pre- and postnatally, given that variations in mitochondrial metabolism can have dramatic impacts on immune and glia cell reactivity. The effects of melatonin seem likely to be integrated with the gut microbiome, especially the levels of butyrate production, which also acts to regulate mitochondrial and immune/glia function. Both butyrate and melatonin act via the regulation of the opioidergic system and therefore with the subjective pleasure and dysphoria that drive food intake.
